# [Retracted] Fundamental research on the action mechanism of the 800 nm semiconductor laser on skin blackheads and coarse pores

**DOI:** 10.3892/etm.2024.12709

**Published:** 2024-09-09

**Authors:** Jie Lin, Li Jing, Hao Zhu, Fu-Sheng Dong

Exp Ther Med 13:235–241, 2017; DOI: 10.3892/etm.2016.3937

Following the publication of this paper, it was drawn to the Editor’s attention by a concerned reader that certain of the western blotting data shown in Fig. 5 on p. 240 were strikingly similar to data appearing in different form in another article written by different authors at different research institutes that had already been published in the journal *Molecular Cancer* prior to the submission of this paper to *Experimental and Therapeutic Medicine*. In view of the fact that the abovementioned data had already apparently been published previously, the Editor of *Experimental and Therapeutic Medicine* has decided that this paper should be retracted from the Journal. The authors were asked for an explanation to account for these concerns, but the Editorial Office did not receive a reply. The Editor apologizes to the readership for any inconvenience caused.

